# The Efficacy of Pulsed Electromagnetic Fields on Pain, Stiffness, and Physical Function in Osteoarthritis: A Systematic Review and Meta-Analysis

**DOI:** 10.1155/2022/9939891

**Published:** 2022-05-09

**Authors:** Jie Tong, Zhengyu Chen, Guanghua Sun, Jun Zhou, Ye Zeng, Peirui Zhong, Chengyuan Deng, Xiaocui Chen, Liu Liu, Shiyong Wang, Jiaqian Chen, Ying Liao

**Affiliations:** ^1^Rehabilitation Medicine Center, The First Affiliated Hospital, Hengyang Medical School, University of South China, Hengyang, Hunan 421001, China; ^2^Department of Rehabilitation, The First Affiliated Hospital, Hengyang Medical School, University of South China, Hengyang, Hunan 421001, China; ^3^Rehabilitation Laboratory, The First Affiliated Hospital, Hengyang Medical School, University of South China, Hengyang, Hunan 421001, China; ^4^Department of Spine, The First Affiliated Hospital, Hengyang Medical School, University of South China, Hengyang, Hunan 421001, China; ^5^Department of Anatomy, Hunan Traditional Chinese Medical College, Zhuzhou, Hunan 421001, China; ^6^Department of Rehabilitation, Affiliated Hospital of Jiangnan University, Wuxi, Jiangsu 214000, China

## Abstract

**Background:**

Although there are many pharmacological interventions for adults with osteoarthritis (OA) who do not meet the indications for surgery, side effects and adverse effects cannot be ignored. Physical interventions are known for their effectiveness and safety, and pulsed electromagnetic fields (PEMFs) have already been applied to skeletal diseases such as osteoporosis.

**Objective:**

In this systematic review and meta-analysis, we aimed to assess the efficacy of PEMF on the major symptoms of patients with OA compared with efficacy of other interventions.

**Methods:**

Randomized controlled trials (RCTs) investigating OA patients treated with PEMF and with pain, stiffness, and physical function impairment since 2009 were included. The Visual Analog Scale (VAS) and Western Ontario McMaster Universities Osteoarthritis Index (WOMAC) scores were used for assessment. All extracted data were analyzed using RevMan V.5.3.

**Results:**

Eleven RCTs consisting of 614 patients were enrolled in this meta-analysis, of which 10 trials comprised knee OA and one comprised hand OA. Compared with the control groups, the PEMF treatment yielded a more favorable output. PEMF alleviated pain (standardized mean differences [SMD] = 0.71, 95% confidence interval [CI]: 0.08–1.34, *p* = 0.03), improved stiffness (SMD = 1.34, 95% CI: 0.45–2.23,*p*=0.003), and restored physical function (SMD = 1.52, 95% CI: 0.49–2.55,*p*=0.004).

**Conclusions:**

PEMF therapy ameliorates OA symptoms such as pain, stiffness, and physical function in patients compared to other conservative treatments. There is an urgent need to search for different types of OA in multiple locations.

## 1. Introduction

Osteoarthritis (OA) is the most frequent type of arthritis, affecting 250 million people worldwide [1]. This type of chronic and relapsing disease causes notable economic costs for both individuals suffering from it and the government [2]. OA is the most common cause of joint destruction, soreness, and movement dysfunction [3]. Insufficient treatment to alter the progression of this disease fails to manage OA [4]. Authentic guidelines have approved the application of interventions for knee OA, including disease education, weight management, and physical treatment [5].

Complementary and alternative medicine, dating back to ancient times [6], has provoked a heated interest in various diseases. Among them, traditional Chinese meditation like Qigong is a mindfulness practice characterized by interlacement of body movements and coordination of body posture, which exposes feasible effects on depressive symptoms, quality of life, and fatigue [7]. In addition, foot reflexology, which applies pressure on the skin to stimulate exact reflexes and transmits them to the central nervous system, helps endocrine function and enzyme hemostasis [8]. Pulsed electromagnetic field (PEMF) is viewed as a safe, noninvasive, and effective physical medicine and is a potential treatment for multiple diseases, including delayed wound healing and OA [9]. A recent study indicated that PEMF not only provides electrical stimulation piezoelectric scaffolds to transport partial mechanical impulses but also provides other functions of long-instance electrical impulses of cell proliferation and differentiation without partial potential of hydrogen alteration or reactive oxygen species creation [10].

Electromagnetic fields (EMFs) ranging from frequencies of 5 Hz to 200 kHz cause an electrical field between 1 and 100 mV/cm in the tissue [11]. The extremely low-frequency electromagnetic field (ELF-EMF) is defined as a field with a frequency of 0–300 Hz mainly produced by coils [12]. Moreover, preclinical in vivo studies have shown that low-frequency PEMF could accelerate Achilles tendon repairment in rats [9]. Apart from variable frequencies, the treatment time, waveform, and amplitude differences in EMF result in various outcomes; thus, there are no authentic recommendations for clinical application [11]. Previous meta-analyses revealed that short-term PEMF therapy (no more than 30 min) could eliminate pain and enhance physical ability for patients who suffer from hand or knee OA more efficiently [13] and have little difference from additional physical interventions such as physiotherapy, transcutaneous electrical nerve stimulation (TENS), hyperthermia, or ultrasound [14], while the main influencing factors, such as frequency of PEMF, have not been demonstrated.

The main goal of this review was to determine the efficacy of PEMF in OA in terms of soreness, stiffness, and physical function. Additionally, we further established subgroups based on frequency to see if both low and high applications are equally beneficial or if some have greater effects than other treatments.

## 2. Methods

This meta-analysis was performed based on the Cochrane Collaboration methodology and PRISMA guidelines [15].

### 2.1. Data Sources

Our protocol has already been registered on PROSPERO websites for systematic reviews (CRD42021288268). Four electronic databases (PubMed, EMBASE, Web of Science, and Cochrane Library) were searched for randomized controlled trials (RCTs) that were conducted from January 1, 2009, to November 1, 2021, and correlated with OA and electromagnetic interventions. The search strategies are presented in the supplementary tables.

### 2.2. Study Eligibility Criteria

Study inclusion criteria were as follows: (1) subjects diagnosed with symptomatic or radiographic OA; (2) PEMF chosen as the intervention as opposed to other treatments or placebo; (3) RCTs; and (4) the main outcomes included pain, stiffness, and/or function assessed by Visual Analog Scale (VAS) and/or Western Ontario McMaster Universities Osteoarthritis Index (WOMAC). Exclusion criteria were as follows: (1) nonclinical trials; (2) patients with other diseases that affect pain, stiffness, and physical function; (3) studies without the full text; (4) studies not written in English; and (5) studies with no comprehensive data available.

### 2.3. Selection Process and Quality Assessment

Two authors extracted all data separately. Basic features of the subjects, including age, sex, duration of OA, protocol treatment of the control group, parameters of PEMF, and baseline, and posttreatment outcomes were derived. The outcomes of pain, stiffness, and physical function are presented as the mean ± standard deviation (SD). The WOMAC and VAS scores were the recommended measures for the outcomes mentioned, and *p* < 0.05 was considered as statistical significance.

### 2.4. Risk of Bias Assessment

**Figure 1:**
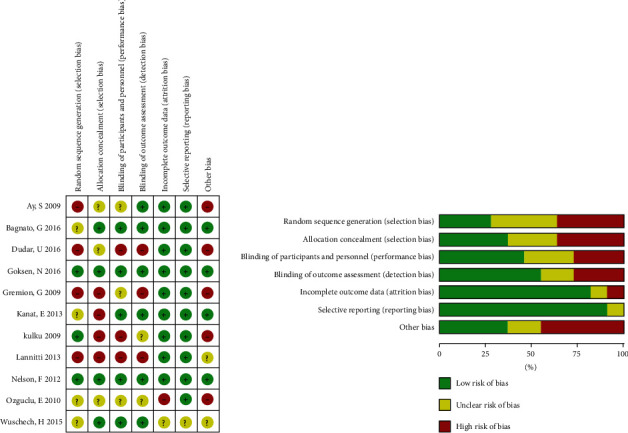
Risk of bias summary of records in this review.

The risk of bias for all studies was measured by two individuals independently, and a third party was prepared in case of divergence. According to the Cochrane Handbook guidelines, a “high,” “unclear,” or “low” risk of bias will be labeled via the judgement of the following seven domains: generation of the random sequence, allocation concealment, blinding of caregivers, patients and outcome assessors, incomplete outcome data collection, and selective reporting [16]. Each domain was assigned a “+” (low risk of bias, with a score of 0), a “?” (unclear risk of bias, with a score of 1), or a “–” (high risk of bias, with a score of 2). A total score of 0–2 indicates a low risk of bias (high quality), 3–5 indicates a moderate risk of bias, and 6–8 indicates a high risk of bias [17]. The risk of bias is presented in (Figure 1).

### 2.5. Investigation of Heterogeneity and Inconsistency

Q-statistics and I^2^ tests were used to assess the heterogeneity and inconsistency of the pooled studies. Significant heterogeneity was considered for *p* > 0.01[18] and I^2^ < 25%, low for I^2^ ≥ 25% *p* > 0.01moderate for I^2^ ≥ 50%, and substantial for I^2^ ≥ 75% [19]. Therefore, a random effect model was selected for assessment of the outcome.

### 2.6. Quantitative Data Analysis

Review Manager V.5.3 (the Cochrane Collaboration, Oxford, UK) was selected for the statistical analyses. Changes in pain, stiffness, and physical function between different therapy groups were measured using standardized mean differences (SMDs) with various scales. A 95% confidence interval (CI) was used to evaluate the overall authenticity of each outcome. It is hypothesized that both the frequency of PEMF and the type of control group violate the impartial assessment. Therefore, subgroup analyses were performed according to the different frequencies of PEMF therapy (no more than 300 Hz or more than 300 Hz). Simultaneously, sham (sham PEMF) and nonblank group (sham PEMF + other alternative therapies) were performed in subgroup analyses.

## 3. Results

### 3.1. Selection of Studies

**Figure 2:**
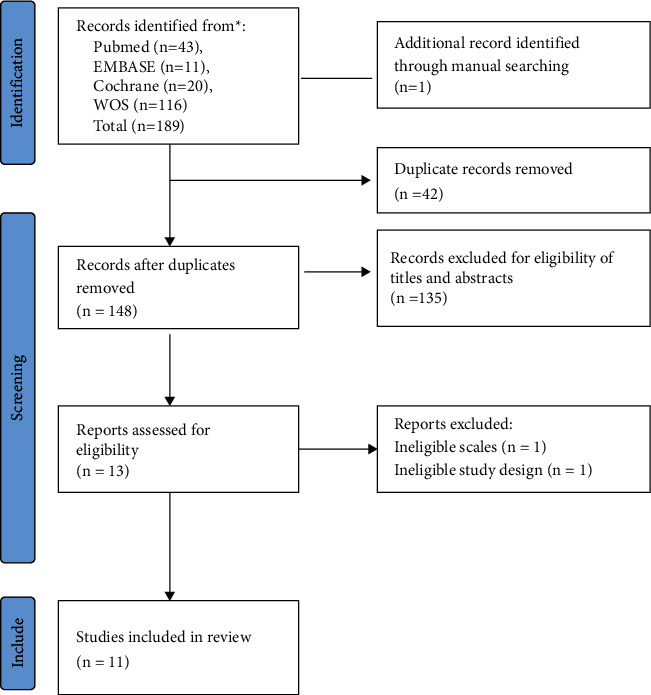
Flow diagram of selection progress for studies.

Identification and screening were applied to 189 records in the database search, one record was manually searched, and 42 duplicates were discarded. As is shown in Figure 1, 13 reports met the inclusion criteria, and two were excluded for ineligible scales and study design after full text screening. A total of 11 records were selected for this meta-analysis, and the selection process is shown in Figure 2.

### 3.2. Characteristics of Included Studies

**Figure 3:**
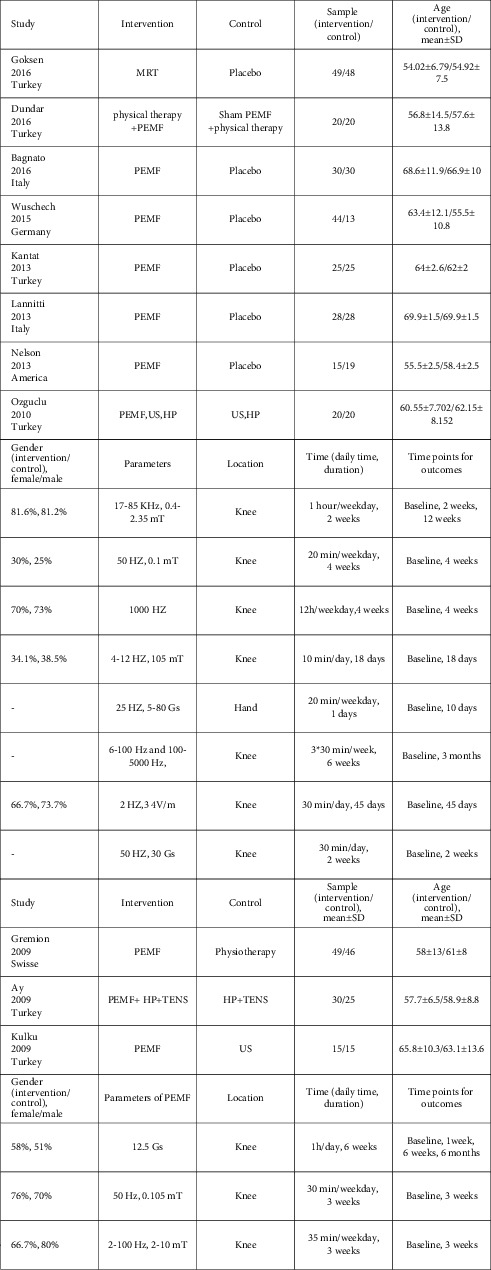
Characteristic of selected records in this meta-analysis. Abbreviations: HP: hot pack, TENS: transcutaneouselectrical nerve stimulation, US: ultrasound, and MRT: magnetic resonance therapy.

Eleven RCTs were conducted in OA patients, with over half conducted in Turkey; two in Italy; and one each in America, Germany, and Switzerland. Pain was recorder for 325 subjects in the PEMF group and 289 subjects in the control group using VAS and/or WOMAC. Six of the 11 studies set a placebo group with an inactive electromagnetic field generator, while five studies set different combinations of alternative methods: hot pack (HP) [20–22], transcutaneous electrical nerve stimulation (TENS) [21, 22], physiotherapy [22, 23], and ultrasound [20, 22, 24].  Figure 3 summarizes the PEMF parameters used in these studies.

### 3.3. Risk of Bias Evaluation in Included Studies

Six studies were at high risk of bias [20–25]and two were at concern for a risk of bias [26, 27], while three were evaluated to have a low risk of bias [28–30]. Among all, the bias risks were predominantly for “allocation concealment” and “random sequence generation.” Furthermore, little evidence of publication bias was found in the evaluation of the funnel plot asymmetry.

### 3.4. PEMF on Pain Relief

**Figure 4:**
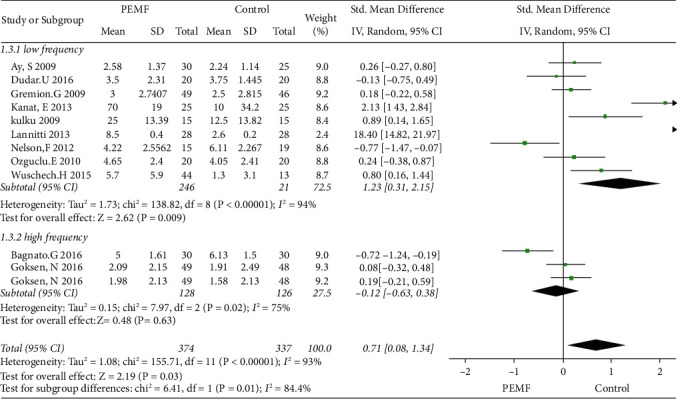
Forest plots representing the effect of PEMF versus control therapies in pain and subgroup analysis basing on various frequency.

**Figure 5:**
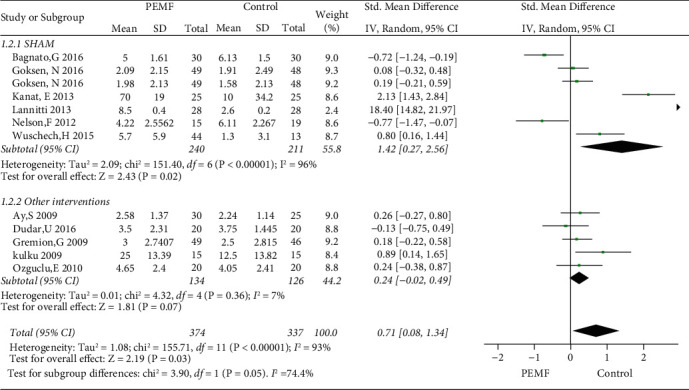
Forest plots representing the effect of PEMF versus control therapies in pain and subgroup analysis basing on different type of control intervention.

**Figure 6:**
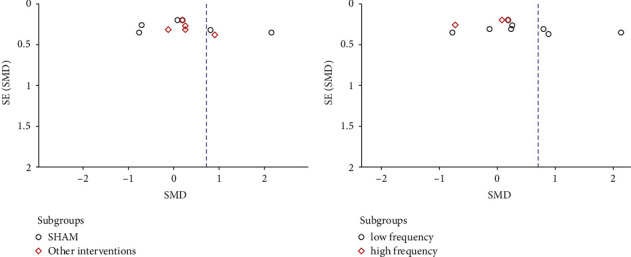
Funnel plot records divided by frequency and type of controls.

Eleven eligible studies regarding pain management were included in this meta-analysis. As shown in Figure 4, compared with the control group, the PEMF group achieved a significant decrease in pain generally (SMD = 0.71, 95% CI: 0.08–1.34,*p*=0.03), with a high heterogeneity (I^2^ = 93%; *p*=0.03). Additionally, subgroup analysis, in terms of frequency, showed that significant differences were observed between the low-frequency PEMF and control therapies in alleviating pain (SMD = 1.23, 95% CI: 0.31–2.15,*p*=0.009), whereas no significant difference was achieved in the high-frequency PEMF group (SMD = -0.12, 95% CI: -0.63–0.38,*p*=0.63). As shown in Figure 5, regarding subgroup analysis concerning with type of control groups, a significant difference was observed in PEMF group compared with the sham PEMF group (SMD = 1.42, 95% CI: 0.27–2.56, *p*=0.02), while no significant difference was achieved when compared to nonblank control group (SMD = 0.24, 95% CI: −0.02–0.49,*p*=0.07) and had low heterogeneity (I^2^ = 7%). Moreover, the funnel plot showed no obvious asymmetry (Figure 6).

### 3.5. PEMF on Stiffness Amelioration

**Figure 7:**
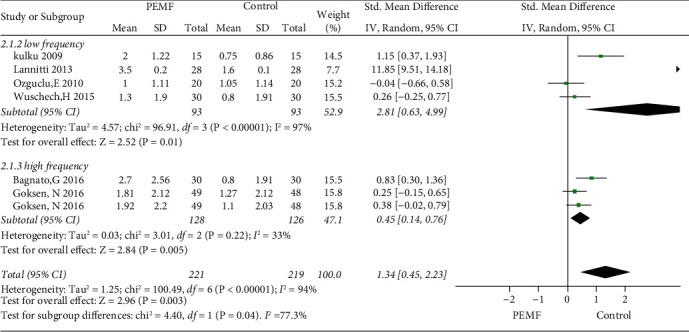
Forest plots represent the effect of PEMF versus control therapies in stiffness and subgroup analysis basing on various frequency.

**Figure 8:**
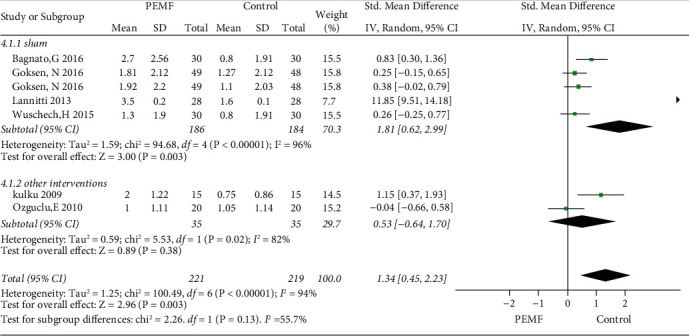
Forest plots represent the effect of PEMF versus control therapies in stiffness and subgroup analysis basing on different type of control intervention.

Six RCTs were included in the analysis of stiffness amelioration. The critical role of PEMF in stiffness improvement is illustrated in Figure 7 (SMD = 1.34, 95% CI: 0.45–2.23,*p*=0.003), with substantial heterogeneity (I^2^ = 99%;*p* < 0.001). The subgroup analysis of variable frequency indicated significant differences in both low or high frequency (SMD = 2.81, 95% CI: 0.63–4.99, *p*=0.01and SMD = 0.45, 95% CI: 0.14–0.76, *p* = 0.005, respectively), compared to sham group. The heterogeneity of the low-frequency subgroup was considerable at 97%, while the high-frequency subgroup had lower heterogeneity at 33%. In the subgroup analysis of various treatments in the control groups, a significant difference was observed with the sham group (SMD = 1.81, 95% CI: 0.62–2.99,*p*=0.003), while a significant difference was not achieved in nonblank control group (SMD = 0.53, 95% CI: −0.64–1.70,*p*=0.38) (Figure 8). Substantial symmetry was observed in the funnel plot (Figure 3).

### 3.6. PEMF on Physical Function

**Figure 9:**
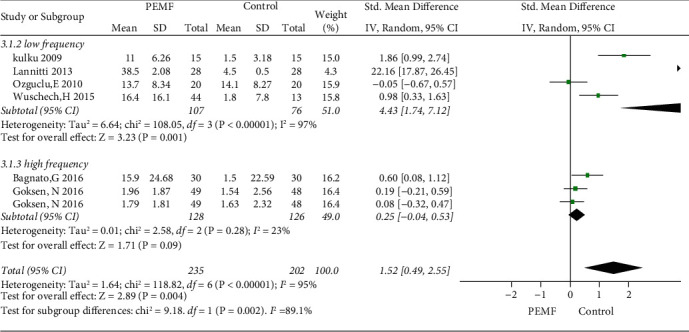
Forest plots represent the effect of PEMF versus control therapies in physical function and subgroup analysis basing on various frequency.

**Figure 10:**
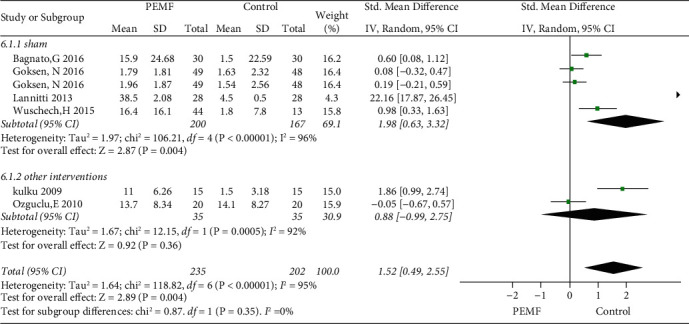
Forest plots represent the effect of PEMF versus control therapies in physical function and subgroup analysis basing on different type of control intervention.

Five RCTs were included in this meta-analysis for the improvement of physical function.  Figure 9 illustrates the role of PEMF in the improvement of physical function in the overall analysis (SMD = 1.52, 95% CI: 0.49–2.55,*p*=0.004) and exhibited substantial heterogeneity (I^2^ = 95%; *p*=0.004). In addition, the subgroup analysis of variable frequency showed a significant difference in function restoration in the low frequency groups (SMD = 4.43, 95% CI: 1.74–7.12,*p*=0.001), whereas there was no significant difference in high frequency groups (SMD = 0.25, 95% CI: -0.04–0.53,*p*=0.09). In addition, a significant difference was observed in the sham group for function enhancement (SMD = 1.98, 95% CI: 0.63–3.32,*p*=0.004) (Figure 10). However, no significant difference was observed in the nonblank control groups (SMD = 0.88, 95% CI: −0.99–2.75,*p*=0.36). Substantial symmetry was observed in the funnel plot (Figure 3).

## 4. Discussion

In this study, we addressed a comprehensive analysis of these scientific records on the influence of the PEMF intervention in patients who suffer from knee or hand OA. A strong relationship between PEMF and OA has been reported in previous literature. Learning from the results above, PEMF showed critical role compared to placebo in pain relief, stiffness restriction, and function enhancement in subjects with knee or hand OA. Though PEMF is not considered for the best choice in treatment of OA, it indeed exerts beneficial effects. This finding provides relatively strong evidence for the clinical application of PEMF in OA.

PEMF, a nonintrusive, safe, and uncomplicated therapy, has been broadly applied to soft tissue impairment fixation, bone fracture treatment, pain alleviation, and inflammation elimination [31]. Recently, researchers have reported that low-frequency PEMF treatment has a significant effect on multiple skeletal diseases such as bone impairment, bone loss, vertebral fusion, and osteoarthritis [32]. In addition, PEMF also prevents degenerative changes in pig knee cartilage [33]. Other studies have shown that high-frequency pulsed electromagnetic fields (HF-PEMFs) could accelerate mineralization and consolidation of bone, upregulate osteogenesis markers [34], and augment osteopontin and osteocalcin expression [35]. In addition, Huegel considered that different frequencies of PEMF treatment will play a positive role in rat rotator cuff rehabilitation [36]. Previous meta-analyses in this field have mainly focused on joint stiffness and physical function without observing the relationship between PEMF parameters and symptoms [37].

Thus, in this systematic review, 11 studies, including 614 patients with OA, were conducted to determine the exact effect of PEMF, on critical symptoms such as pain, stiffness, and physical function. From a global perspectivethe results showed that pain indicators such as WOMAC and VAS dropped significantly compared with baseline. Furthermore, WOMAC scores for stiffness and physical function also revealed substantial outcomes compared to control interventions. The symptoms of patients with OA evaluated with Lattinen scores, based on the exclusion criteria, were abandoned [38]. Simultaneously, researchers compared PEMF with physical therapies and obtained strikingly varied results. In one study, physical methods such as short waves and TENS were included as a control group and compared to PEMF in OA patients and exhibited better pain relief [22], whereas Ayet al. derived opposite results with a longer duration [21]. An in vitro study, performed for 14 days in bone marrow stem cells with a 0.4 T magnetic field, illustrated the role of the magnetic field in the differentiation of chondrocytes through a TGF-*β*-dependent pathway [39]. Another in vivo study pointed out the impact of PEMF on chondrogenic proliferation, differentiation, and extracellular matrix composition via the secretion of anabolic factors, including bone morphogenetic proteins and anti-inflammatory cytokines [40].

Regarding the type of control and frequency that may pose a risk of bias, we categorized subgroups based on parameters such as frequency in PEMF for OA and found that PEMF generally provided positive effects at both low and high frequencies. Stunningly, prior research insisted that, although with relatively low quality, dynamic electricity stimulus under 100 Hz is beneficial in physical ability improvement but not in pain alleviation in OA subjects [41]. Specifically, in our view, lower frequencies performed better in pain amelioration, stiffness restriction, and daily activity enhancement. Moreover, the high-frequency group failed to show significance in pain remission and physical function enhancement. However, the poor quality of high-frequency PEMF treatment for OA has been attributed to the insufficient number of related studies.

Concerning the bias caused by the type of control treatment, such as extracorporeal shock wave therapy, short wave, and other physical therapy combined with sham PEMF, and subgroups were categorized based on the type of control group which divided into sham and nonblank groups. Unexpectedly, the changes in pain, stiffness, and physical ability in the control group with other alternative therapies were not significant in our subgroup analysis, which is consistent with previous records [14, 42].

The major limitations of this study cannot be disregarded. First, a limited number of high-frequency PEMFs were considered to hinder the comparison between low and high frequencies; thus, the favorable rate needs further investigation. Second, the intensity of PEMF was not discussed in this review because of the lack of statistical data. However, previous studies have treated intensity as the main parameter that affects outcomes [14]. Subsequently, high heterogeneity was observed in our review. Finally, comprehensive exploration is needed in the field of both hand and cervical OA patients who suffer from pain and other symptoms, considering the relatively insufficient concentration given to them. At last, the duration of those selected papers mainly between 1 and 3 weeks, thus the long-term efficiency was urged to be demonstrated and assessed.

## 5. Conclusion

Our review highlights the strength of PEMF in pain alleviation, stiffness remission, and physical function restoration in adults with knee or hand OA. In addition, low-frequency PEMF treatment exerts a more favorable efficacy in pain alleviation, stiffness, and physical function improvement. However, given the insufficient number of records based on high-frequency PEMF for OA, further studies considering the limited number of studies with high frequency treatment will need to be undertaken. Apart from this, the side effects of PEMF treatment were not mentioned or assessed in the selected studies, as safety is the primary issue in further clinical applications. Finally, the duration of PEMF treatment is worthy of deep exploration, considering the long course of OA.

## Data Availability

The research data used to support the findings of this study are included within the article.

## References

[1] Cai G., Aitken D., Laslett L. L. (2020). Effect of intravenous zoledronic acid on tibiofemoral cartilage volume Among patients with knee osteoarthritis with bone marrow lesions. *JAMA*.

[2] Safiri S., Kolahi A. A., Smith E. (2020). Global regional and national burden of osteoarthritis 1990-2017: a systematic analysis of the Global Burden of Disease Study 2017. *Annals of the Rheumatic Diseases*.

[3] Butterfield N. C., Curry K. F., Steinberg J. (2021). Accelerating functional gene discovery in osteoarthritis. *Nature Communications*.

[4] Li T., Chubinskaya S., Esposito A. (2019). TGF-*β* type 2 receptor-mediated modulation of the IL-36 family can be therapeutically targeted in osteoarthritis. *Science Translational Medicine*.

[5] Arden N. K., Perry T. A., Bannuru R. R. (2021). Non-surgical management of knee osteoarthritis: comparison of ESCEO and OARSI 2019 guidelines. *Nature Reviews Rheumatology*.

[6] Mosavat S. H., Ghahramani L., Haghighi E. R., Chaijan M. R., Hashempur M. H., Heydari M. (2015). Anorectal diseases IN AVICENNA’S “canon OF medicine. *Acta Medico-Historica Adriatica : AMHA*.

[7] Leung K. W., Yang Y. J., Hui S. S., Woo J. (2021). Mind-body health benefits of traditional Chinese Qigong on women: a systematic review of randomized controlled trials. *Evid Based Complement Alternat Med*.

[8] Zeidabadinejad S., Mangolian Shahrbabaki P., Dehghan M. (2021). Effect of foot reflexology on sexual function of patients under hemodialysis: a randomized parallel controlled clinical trial. *Evidence-based Complementary and Alternative Medicine : eCAM*.

[9] Gehwolf R., Schwemberger B., Jessen M. (2019). Global responses of il-1*β*-primed 3D tendon constructs to treatment with pulsed electromagnetic fields. *Cells*.

[10] Dong Y., Suryani L., Zhou X. (2021). Synergistic effect of PVDF-coated PCL-TCP scaffolds and pulsed electromagnetic field on osteogenesis. *International Journal of Molecular Sciences*.

[11] Ziegler P., Nussler A. K., Wilbrand B. (2019). Pulsed electromagnetic field therapy improves osseous consolidation after high tibial osteotomy in elderly patients-A randomized placebo-controlled double-blind trial. *Journal of Clinical Medicine*.

[12] Cios A., Ciepielak M., Stankiewicz W., Szymański Ł. (2021). The influence of the extremely low frequency electromagnetic field on clear cell renal carcinoma. *International Journal of Molecular Sciences*.

[13] Strauch B., Patel M. K., Rosen D. J. (2006). Pulsed magnetic field therapy increases tensile strength in a rat Achilles’ tendon repair model. *Journal of Hand Surgery*.

[14] Viganò M., Perucca Orfei C., Ragni E., Colombini A., de Girolamo L. (2021). Pain and Functional Scores in Patients Affected by Knee OA after Treatment with Pulsed Electromagnetic and Magnetic Fields: A Meta-Analysis. *Cartilage*.

[15] Page M. J., McKenzie J. E., Bossuyt P. M. (2021). The PRISMA 2020 statement: an updated guideline for reporting systematic reviews. *Bmj*.

[16] Higgins J. P. T., Altman D. G., Gotzsche P. C. (2011). The Cochrane Collaboration’s tool for assessing risk of bias in randomised trials. *Bmj*.

[17] Fu Z., Burger H., Arjadi R., Bockting C. L. H. (2020). Effectiveness of digital psychological interventions for mental health problems in low-income and middle-income countries: a systematic review and meta-analysis. *The Lancet Psychiatry*.

[18] Consortium C. A. D., Deloukas P., Kanoni S. (2013). Large-scale association analysis identifies new risk loci for coronary artery disease. *Nature Genetics*.

[19] Achten N. B., Klingenberg C., Benitz W. E. (2019). Association of use of the neonatal early-onset sepsis calculator with reduction in antibiotic therapy and safety. *JAMA Pediatrics*.

[20] Ozgüçlü E., Cetin A., Cetin M., Calp E. (2010). Additional effect of pulsed electromagnetic field therapy on knee osteoarthritis treatment: a randomized placebo-controlled study. *Clinical Rheumatology*.

[21] Ay S., Evcik D. (2009). The effects of pulsed electromagnetic fields in the treatment of knee osteoarthritis: a randomized placebo-controlled trial. *Rheumatology International*.

[22] Dündar Ü, Aşık G., Ulaşlı A. M. (2016). Assessment of pulsed electromagnetic field therapy with Serum YKL-40 and ultrasonography in patients with knee osteoarthritis. *International journal of rheumatic diseases*.

[23] Gremion G., Gaillard D., Leyvraz P., Jolles B. (2009). Effect of biomagnetic therapy versus physiotherapy for treatment of knee osteoarthritis: a randomized controlled trial. *Journal of Rehabilitation Medicine*.

[24] Kulcu D. G., Gulsen G., Altunok E. C. (2009). Short-term efficacy of pulsed electromagnetic field therapy on pain and functional level in knee osteoarthritis: a randomized controlled study. *Turk J Rheumatol*.

[25] Iannitti T., Palmieri B., Fistetto A., Esposito V., Rottigni B. (2013). Pulsed electromagnetic field therapy for management of osteoarthritis-related pain stiffness and physical function: clinical experience in the elderly. *Clinical Interventions in Aging*.

[26] Kanat E., Alp A., Yurtkuran M. (2013). Magnetotherapy in hand osteoarthritis: a pilot trial. *Complementary Therapies in Medicine*.

[27] Wuschech H., von Hehn U., Mikus E., Funk R. H. (2015). Effects of PEMF on patients with osteoarthritis: results of a prospective placebo-controlled double-blind study. *Bioelectromagnetics*.

[28] Nelson F., Zvirbulis R., Pilla A. A. (2012). Non-invasive electromagnetic field therapy produces rapid and substantial pain reduction in early knee osteoarthritis: a randomized double-blind pilot study. *Osteoarthritis and Cartilage*.

[29] Goksen N., Calis M., Dogan S., Calis H. T., Ozgocmen S. (2016). Magnetic resonance therapy for knee osteoarthritis: a randomized double blind placebo controlled trial. *European Journal of Physical and Rehabilitation Medicine*.

[30] Bagnato G. L., Miceli G., Marino N., Sciortino D., Bagnato G. F. (2016). Pulsed electromagnetic fields in knee osteoarthritis: a double blind placebo-controlled randomized clinical trial. *Rheumatology*.

[31] Chen Y., Aspera-Werz R. H., Menger M. M. (2021). Exposure to 16 Hz pulsed electromagnetic fields protect the structural integrity of primary cilia and associated TGF-*β* signaling in osteoprogenitor cells harmed by cigarette smoke. *International Journal of Molecular Sciences*.

[32] Ehnert S., Schröter S., Aspera-Werz R. H. (2019). Translational insights into extremely low frequency pulsed electromagnetic fields (ELF-PEMFs) for bone regeneration after trauma and orthopedic surgery. *Journal of Clinical Medicine*.

[33] Tan L., Ren Y., van Kooten T. G., Grijpma D. W., Kuijer R. (2015). Low-intensity pulsed ultrasound (LIPUS) and pulsed electromagnetic field (PEMF) treatments affect degeneration of cultured articular cartilage explants. *International Orthopaedics*.

[34] Oltean-Dan D., Dogaru G. B., Apostu D. (2019). Enhancement of bone consolidation using high-frequency pulsed electromagnetic fields (HF-PEMFs): an experimental study on rats. *Bosnian Journal of Basic Medical Sciences*.

[35] Teven C. M., Greives M., Natale R. B. (2012). Differentiation of osteoprogenitor cells is induced by high-frequency pulsed electromagnetic fields. *Journal of Craniofacial Surgery*.

[36] Huegel J., Choi D. S., Nuss C. A. (2018). Effects of pulsed electromagnetic field therapy at different frequencies and durations on rotator cuff tendon-to-bone healing in a rat model. *Journal of Shoulder and Elbow Surgery*.

[37] Yang X., He H., Ye W., Perry T. A., He C. (2020). Effects of pulsed electromagnetic field therapy on pain stiffness physical function and quality of life in patients with osteoarthritis: a systematic review and meta-analysis of randomized placebo-controlled trials. *Physical Therapy*.

[38] Pavlović A. S., Djurasić L. M. (2012). The effect of low frequency pulsing electromagnetic field in treatment of patients with knee joint osteoarthritis. *Acta chirurgica Iugoslavica*.

[39] Amin H. D., Brady M. A., St-Pierre J. P., Stevens M. M., Overby D. R., Ethier C. R. (2014). Stimulation of chondrogenic differentiation of adult human bone marrow-derived stromal cells by a moderate-strength static magnetic field. *Tissue Engineering. Part A*.

[40] Iwasa K., Reddi A. H. (2018). Pulsed electromagnetic fields and tissue engineering of the joints. *Tissue Engineering Part B Reviews*.

[41] Negm A., Lorbergs A., Macintyre N. J. (2013). Efficacy of low frequency pulsed subsensory threshold electrical stimulation vs placebo on pain and physical function in people with knee osteoarthritis: systematic review with meta-analysis. *Osteoarthritis and Cartilage*.

[42] Ryang We S., Koog Y. H., Jeong K.-I., Wi H. (2013). Effects of pulsed electromagnetic field on knee osteoarthritis: a systematic review. *Rheumatology*.

