# Supplementary material for: The Efficacy of Pulsed Electromagnetic Fields on Pain, Stiffness, and Physical Function in Osteoarthritis: A Systematic Review and Meta-Analysis
Source: Pain Res Manag. 2022 May 9;2022:9939891. doi: 10.1155/2022/9939891 (PMC9110240; doi:10.1155/2022/9939891)
Supplement: Supplementary Materials — The search strategy in this review is provided. [file 9939891.f1.docx]

| Pubmed | # 1 (("Arthritis"[Mesh] OR "Osteoarthritis"[Mesh] OR "Cartilage Diseases"[Mesh]))  # 2 (("Electromagnetic Fields"[Mesh] OR "Magnetic Field Therapy "[Mesh] OR "Electromagnetic phenomena"[Mesh]))  # 3 randomized controlled trial [Publication Type]  # 4 #1 AND #2 AND #3 |
| --- | --- |
| EMBASE | # 1 ‘pulsed electromagnetic field’/exp  # 2 ‘PEMF’  # 3 ‘magnetic field’  # 4 ‘magnetic field therapy’  # 5 #1 OR #2 OR #3 OR #4  # 6 ‘osteoarthritis’/exp  # 7 ‘arthritis’/exp  # 8 #6 OR #7  # 9 ‘randomized controlled trial’/exp  #10 #5 AND #8 AND #9 |
| Web of Science | # 1 TS= (arthritis OR osteoarthritis)  # 2 TI= (magnetic OR electromagnetic OR PEMF)  # 3 ALL=(Randomized)  # 4 (#3 AND #2 AND #1) |
| Cochrane | # 1 TS= (arthritis OR osteoarthritis)  # 2 TI= (magnetic OR electromagnetic OR PEMF OR magnetic field therapy OR electromagnetic phenomena)  # 3 ALL= (randomized)  # 4 #1 AND #2 AND #3 |
